# Novel Selective Butyrylcholinesterase Inhibitors Incorporating Antioxidant Functionalities as Potential Bimodal Therapeutics for Alzheimer’s Disease

**DOI:** 10.3390/molecules21040440

**Published:** 2016-04-01

**Authors:** Mike Jones, Jun Wang, Shona Harmon, Beata Kling, Jörg Heilmann, John F. Gilmer

**Affiliations:** 1 School of Pharmacy and Pharmaceutical Sciences, Trinity College, Dublin 2, Ireland; mike_jone5@hotmail.com (M.J.); wangju@tcd.ie (J.W.); s.harmon@redxpharma.com (S.H.); 2 Institute of Pharmacy, University of Regensburg, 93053 Regensburg, Germany; Beata.Kling@chemie.uni-regensburg.de (B.K.); Joerg.Heilmann@chemie.uni-regensburg.de (J.H.)

**Keywords:** neuroprotection, Alzheimer’s disease, antioxidant, cholinesterase inhibitor, hybrid

## Abstract

Isosorbide-2-carbamates-5-aryl esters are highly potent and very selective butyrylcholinesterase inhibitors. The objective of the present work was to address the hypothesis that the isosorbide-aryl-5-ester group could be replaced with an antioxidant functionality while maintaining inhibitor effects and selectivity. We successfully incorporated ferulic acid or lipoic acid groups producing potent selective inhibitors of butyrylcholinesterase (BuChE). The hybrid compounds were non-toxic to the murine hippocampal cell line HT-22 and lipoate esters were neuroprotective at 10 and 25 µM when the cells were challenged with glutamate (5 mM) in a similar manner to the positive control quercetin. The benzyl carbamate **7a** was a potent inhibitor of BuChE (IC_50_ 150 nM) and it was effective in reducing glutamate toxicity to neuronal cells at >5 µM. Representative compounds exhibited an antioxidant effect in the oxygen radical absorbance capacity assay as the lipoate **7d** was not active, whereas the ferulate **8a** showed a weak, but significant, activity with 0.635 ± 0.020 Trolox Equivalent.

## 1. Introduction

Alzheimer’s disease (AD) is now the most common neurodegenerative disease, with more than 20 million cases worldwide [1]. AD is characterised by global cognitive decline and associated neuropathological findings which may include neuronal loss, neurofibrillary tangles, neuritic plaques and amyloid angiopathy. Cholinergic loss is the single most replicated neurotransmitter deficiency in AD, and AChE has been a drug target for the treatment of AD since the emergence of this cholinergic hypothesis over 30 years ago [2]. This followed recognition that the cognitive impairments in AD correlated with cholinergic deficits such as reduced synaptic acetylcholine synthesis and choline acetyltransferase (ChAT) activity. The introduction of the cholinesterase inhibitors (ChEIs)—first tacrine then donepezil, rivastigmine, and galantamine—has made an important contribution to the management and well-being of early stage AD patients. Selective inhibitors of acetylcholinesterase (AChE; EC 3.1.1.7) e.g., donepezil and unselective inhibitors (ChEIs) e.g., rivastigmine, induce dose-limiting adverse effects such as bradycardia, nausea, and diarrhea [3,4].

There are challenges facing this field in identifying new agents that have improved efficacy or side effect profile. Medicinal chemistry themes that can be recognised as a response to this include a switch in focus from AChE to butyrylcholinesterase (BuChE; EC 3.1.1.8) [5,6,7]. This is because of the observations that BuChE assumes a more important role in the AD brain during disease progression [8,9,10]. Another major theme has been the design of hybrids able to modulate simultaneously several aspects of the complex Alzheimer pathobiology [11,12]. There is a lot of current interest, for example, in the design of hybrid compounds able to inhibit cholinesterase and attenuate neuronal apoptosis associated with tissue damage and reactive oxygen species [13]. The antioxidant system loses effectiveness during the aging process and oxidative damage has been observed before the formation of AD-specific pathological β-amyloid plaques [14]. α-Lipoic acid is an interesting substance in this context because it plays an essential role in mitochondrial dehydrogenase reactions. Lipoate, or its reduced form, dihydrolipoate, reacts with reactive oxygen species such as superoxide radicals, hydroxyl radicals, hypochlorous acid, peroxyl radicals, and singlet oxygen [15]. α-Lipoate is taken up and reduced in cells and tissues to dihydrolipoate. Dihydrolipoate is also exported to the extracellular medium; therefore, protection is afforded to both intracellular and extracellular environments. Thus, α-lipoate would seem an ideal substance in the treatment of oxidative brain and neural disorders involving free radical processes. Lipoic acid has previously been linked to the ChI compounds tacrine and quinazolinimines producing potential bimodal antioxidant cholinesterase inhibitory therapeutics for AD [16,17]. The introduction of LA into ChIs has also been used to alter and considerably improve ChE inhibition and isoenzyme selectivity [16,17]. Ferulic acid (FA) and its ester derivatives are also attractive candidates for incorporation into a hybrid design. These are polyphenolic compounds with potent antioxidant activity *in vitro*, due to their ability to scavenge radicals [18]. They have also been shown to up-regulate cytoprotective enzymes such as heme oxygenase-1 and heat shock protein 70, and subsequently inhibit oxidative stress and cell death in neurons treated with beta amyloid peptide [19]. Pretreatment with ferulic acid in a mouse model was also shown to inhibit oxidative damage induced memory deficit *in vivo* [20]. This evidence, taken together, suggested that ester derivatives of ferulic acid could be brain accessible, multifunctional compounds for the treatment of AD. Several examples have been reported of successful incorporation of ferulic acid into ChIs [21].

**Figure 1:**
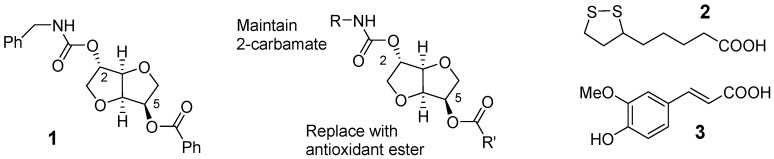
Isosorbide-based carbamates e.g., **1** are potent selective inhibitors of BuChE; formulae for lipoic acid (**2**) and ferulic acid (**3**).

The present study concerns the design of neuroprotective hybrids that could selectively inhibit BuChE over AChE and modulate ROS damage associated. Our design for accomplishing BuChE specific inhibitors with additional anti-oxidant activity relies on observations around some carbamates we have reported previously. Isosorbide-2-alkyl and aryl carbamates are selective and potent inhibitors of BuChE (e.g., **1** in Figure 1, IC_50_ BuChE 4.3 nM), and they are selective for BuChE over AChE and related carboxylesterases including CE1 and CE2 found in human liver and intestine [22]. Whereas the 2-carbamate is essential to activity, we showed that the 5-position of the isosorbide scaffold could be modified in diverse ways producing more or less potent compounds [23]. We hypothesised that it might be possible to retain the inhibitory isosorbide-2-carbamate functionality but substitute the effective 5-benzoate in **1** with lipoic acid or ferulic acid esters, retaining cholinesterase sub-type selectivity while introducing new disease modifying functionality as shown in Figure 1.

## 2. Results and Discussion

### 2.1. Synthesis of Isosorbide-Based Carbamate-Antioxidants

Isosorbide-2-carbamate-5-nitrates (**5a**–**d**) were obtained by direct carbamylation of isosorbide mononitrate with the appropriate isocyanate in pyridine. The 2-carbamates **6a**–**d** were obtained by removal of the nitrate group under reductive conditions (H_2_, Pd/C). For this work, we chose benzyl carbamate because, in the isosorbide-2-carbamate-5-ester compounds already reported [22], benzyl carbamates were most potent irrespective of the identity of the 5-ester. Indeed the most potent compound we reported was a 2-benzyl carbamate with a 5-salicylate group which had an IC_50_ for BuChE of <150 pM and an estimated 500,000 fold selectivity over AChE [24]. To investigate the present design hypothesis, we also chose butyl and ethyl carbamates because these are also active in isosorbide-5-ester cases, depending on the 5-ester identity. 

**Scheme 1:**
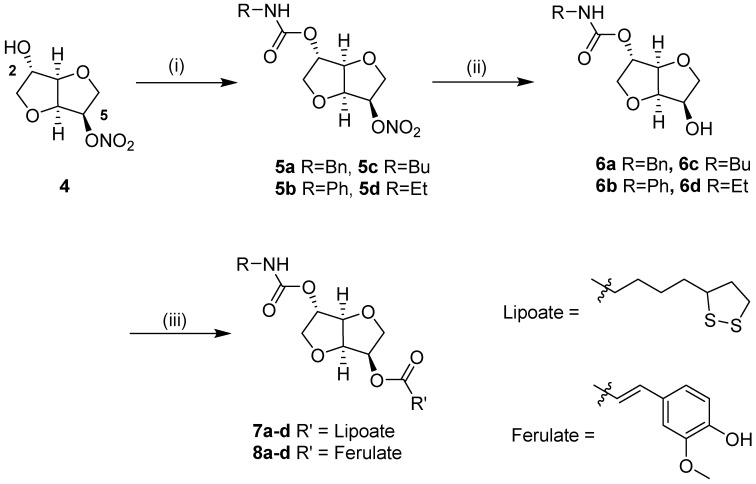
Synthesis of hybrids **7a**–**d** and **8a**–**d**. (i) RNCO, pyridine 100 °C, 2 h; (ii) Pd/C, H_2_, EtOAc, 12 h, RT; (iii) R’C(O)OH, EDCI, DMAP, DCM, RT, 16 h.

Isosorbide-based phenyl carbamates tend to be more AChE than BuChE selective, so we included a phenyl carbamate in this series to see if the pattern would be maintained and to diversify the expected pharmacology of the product hybrid compounds. Esterification of **6a**–**d** with lipoic acid produced **7a**–**d**; esterification with ferulic acid afforded **8a**–**d**. Esterification was achieved using standard EDCI/DMAP coupling (Scheme 1).

### 2.2. Cholinesterase Inhibition and Mechanism

Inhibitory activities of the isosorbide-2-carbamates bearing a lipoate ester (**7a**–**d**), a ferulate ester (**8a**–**d**) ester or 5-unsubstituted compounds (**6a**–**d**) was measured using the standard Ellman spectrophotometric method using AChE from electric eel or BuChE purified from human plasma with acetylthiocholine or butyrylthiocholine as appropriate (Table 1). The most potent compounds across the three series were the 5-lipoates **7a** and **7c**, which were 2-benzyl and -butyl carbamates. These were potent, selective inhibitors of BuChE (IC_50_, 150 and 170 nM, respectively). The lipoate esters were in each case more potent that the ferulate esters. The most potent 5-ferulate was the butyl carbamate **8c** (BuChE IC_50_, 430 nM). These values are in the potency range of clinically used cholinesterase inhibitors. None of the hybrid compounds exhibited notable inhibitory effects against AChE, apart from **8b**, the phenyl carbamate which was a moderately potent inhibitor (IC_50_ 29.16 µM). In most cases, it was not possible to achieve a maximal inhibition of AChE because the solubility threshold was lower than the concentration required for maximal inhibition. In previous studies, we noticed that phenyl carbamates of isosorbide are relatively weak inhibitors of BuChE compared with the benzyl and alkyl carbamates, but that AChE is relatively tolerant of isosorbide phenyl carbamates. We published a paper showing that isosorbide-2,5-diaryl esters are moderately potent substrate-inhibitors of AChE [25]. Both types of 5-ester in the present study—lipoate and ferulate—were more potent than the corresponding unsubstituted compounds **6a**–**d**. The effect of (appropriate) 5-ester substitution in the series of isosorbide compounds already published is to increase potency by promoting affinity in the pre-carbamylation state, reducing the pseudo Michaelis constant for the carbamate substrate (referred to as *K*_c_ in carbamylation literature) [24]. When the intrinsic affinity of the substrate carbamate is higher, there is a relatively greater proportion of the unimolecular *E. carbamate* complex. In the present case, the anti-oxidant ester functionality directly contributes to potency by increasing substrate affinity. The greater potency of the alkyl lipoate ester relative to the aromatic ferulate was unexpected because in previous isosorbide-carbamate series, 5-aryl ester carbamates were more potent. In the *in vivo* situation, the intact carbamate might be expected to exhibit antioxidant properties, or the isosorbide-5-ferulate or lipoate product of carbamylation. Indeed, ester hydrolysis at the isosorbide C-5, which is slow, would produce the ferulic acid or lipoic acid byproduct. The compounds may therefore be classified as antioxidant acid prodrugs or, because they are more active intact, carbamate-soft drugs. 

The time dependence of BuChE inhibition associated with the pseudoirreversible carbamylation phase was investigated for the most potent BuChE inhibitor **7a**. Solutions of human BuChE were treated with **7a** in the range of 1–50 µM. At successive time intervals, aliquots were removed and the carbamylation reaction stopped by dilution. Residual enzyme activity at each time point and each inhibitor concentration level was estimated by adding the sample to a solution containing butyrylthiocholine substrate. As expected, enzyme activity decayed time and concentration dependently with pseudo first order rate constants at 50 µM of 0.198 min^−1^ (r^2^ > 0.99) and at 10 µM 0.068 min^−1^ (r^2^ > 0.99). These are in the expected range for cholinesterease carbamylation indicating that pseudo irreversible covalent bond formation occurred. 

### 2.3. Neuroprotection and Antioxidant Activity

The neuroprotective properties of the compounds were assessed using the HT-22 murine hippocampal cell line. This is an immortalized cell line which has become widely used in recent years to study oxidative stress induced neuronal cell death. The cell line lacks ionotropic glutamate receptors but is sensitive to glutamate cytotoxicity via non-receptor mediated pathway(s) that are to a significant extent propagated by reactive oxygen species [26,27]. The cell line appeared to be a good cellular model for measuring an anti-oxidant mediated neuroprotective *in vitro* effect of the compounds [26,27,28].

**Figure 2:**
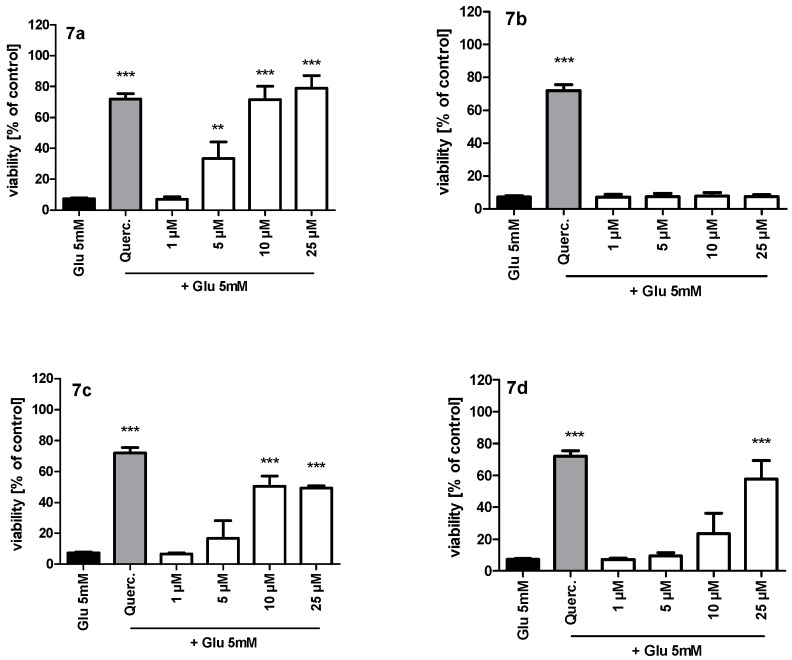
Neuroprotection was investigated by treating HT-22 cells in culture with glutamate (5 mM) and lipoates **7a**–**d** in the range 1–25 µM (*n =* 3, means ± SD); quercetin (25 µM) was used as positive control. One-way ANOVA followed by Dunnett’s multiple comparison post-test using GraphPad Prism 5 Software. (Levels of significance ** *p* < 0.01; *** *p* < 0.001).

Test compounds in DMSO solution (0.1%) were added to HT-22 cell culture for (24 h), using four repeats in three independent experiments. Some solubility issues were observed at 50 µM in culture medium with the ferulate ester series. These compounds also caused a small reduction in cell viability in the range 1–25 µM which was significant only in the case of **8d** at 25 µM (data not shown). The lipoates did not manifest solubility problems and did not cause any reduction in cell viability when tested by themselves, indicating a lack of intrinsic neurotoxicity. Lipoic acid and ferulic acid were also non-toxic in the same concentration range. Next, the neuronal cell culture was treated with glutamate (5 mM) which caused about 90% cell death as reflected in the MTT assay. Quercetin served as positive control and caused rescue of cell death to around 80% cell viability at 25 µM. Cells were co-treated with test compounds **7a**–**d** and **8a**–**d** at 1, 5, 10 and 25 µM in four repeats and three independent experiments. Lipoic and ferulic acid were tested in parallel experiments in the same concentration range. Lipoic acid was active in the range 10–25 µM but ferulic acid was not active (data not shown). Several compounds in the lipoate series (**7a**–**d**) produced a concentration dependent effect as shown in Figure 2. The most effective compound was the benzyl carbamate **7a**, which had also been the most potent and selective BuChE inhibitor. This was effective in the range 5–25 µM, slightly more effective than lipoic acid, which was not effective at 5 µM and similar in efficacy to the positive control quercetin at 25 µM. Surprisingly, a neuroprotective effect could not be observed during treatment with the AChE phenyl carbamate **7b**. The butyl and ethyl carbamates **7c** and **7d** showed some effects but they were not as potent or efficacious as the benzyl carbamate. The reasons for this unexpected variation in efficacy is not clear but may be due to (intra)cellular access. Ferulic acid and the isosorbide-based ferulate ester series **8a**–**d** did not exhibit any protective effects in this model in the range 1–25 µM.

The antioxidant capacity of **7d** and **8a** was assessed directly using the oxygen radical absorbance capacity (ORAC) assay, in which the physicochemical ability of the test compounds to scavenge radicals and quench fluorescein absorbance is quantified and related to so-called trolox equivalents (TE = 1 represents the antioxidant equivalence to trolox, a water-soluble Vitamin E derivative) [6,16,29,30]. Compound **7d** was 0.163 ± 0.146, **8a**, 0.635 ± 0.020, thus showing a very weak ORAC for the lipoate and a weak, but significant, effect for the ferulic acid derivative. The lack of activity of the lipoate in the non-cellular ORAC assay is not surprising as its anti-oxidant effects are mainly related to cellular or mitochondria related processes. 

## 3. Materials and Methods 

### 3.1. General Chemistry

^1^H and ^13^C spectra were recorded at 27 °C on a Bruker DPX 400 MHz FT NMR spectrometer (400.13 MHz ^1^H, 100.61 MHz ^13^C) or a Bruker AV600 (600.13 MHz ^1^H, 150.6 MHz ^13^C, Bruker, Coventry, UK). In CDCl_3,_^1^H spectra were assigned relative to the TMS peak at 0.0 ppm, and ^13^C spectra were assigned relative to the middle CDCl_3_ triplet at 77.00 ppm. Coupling constants were reported in hertz (Hz). HRMS (positive mode) was performed using a Micromass mass spectrophotometer (Waters, Herdfordshire, UK) with electrospray ionization at the School of Chemistry, Trinity College Dublin (Dublin, Ireland).

#### 3.1.1. General Procedure for Synthesis of Isosorbide-2-carbamates-5-nitrates

The 2-carbamate-5-nitrates were prepared as described.^22^ Isosorbide mono-nitrate (ISMN), (0.5 g, 2.6 mmol, 1 eq.) was dissolved in anhydrous pyridine (5 mL). Appropriate isocyanate (2 eq.) was added and the reaction mixture was heated to 100 °C for 1 h, in a round bottom flask equipped with a reflux condenser. Methanol (3.5 mL) was then added and the reaction was continued for ten minutes to remove excess isocyanate. After this time, the reaction mixture was cooled to room temperature and dropped onto ice-water. The resulting precipitate was removed by filtration, washed with ice-water and recrystallised using ethanol to yield pure isosorbide-2-carbamate-5-nitrate. Spectroscopic and chemical properties of **5a**–**d** were as reported [22].

#### 3.1.2. General Procedure for the Preparation of Isosorbide-2-carbamates (GP2)

*Isosorbide-2-benzylcarbamate* (**6a**)*.* Isosorbide-2-benzylcarbamate-5-nitrate (0.5 g, 1.5 mmol) was dissolved in 100 mL of a 1:1 mixture of ethyl acetate and methanol in a round bottom flask and palladium on activated carbon (50 mg, 10% *w*/*w*) was added. Air was expelled from the flask and the reaction mixture was stirred under an atmosphere of hydrogen for 24 h. After this time palladium removed by filtration through a pad of celite, solvent was removed under reduced pressure and pure (**5**) was recrystallised from ethanol as white crystals (0.428 g, 100%). mp 76.0 °C. Spectroscopic features were as described previously [22].

*Isosorbide-2-phenylcarbamate* (**6b**)*.* Yield 100%. ^1^H-NMR (400 MHz, CDCl_3_) δ 7.4 (4H, m), 7.1 (1H, t, *J =* 7.5 Hz), 6.8 (1H, br s), 5.3 (1H, d, *J =* 3 Hz), 4.7 (1H, t, *J =* 5 Hz), 4.5 (1H, d, *J =* 3 Hz), 4.3 (1H, q, *J =* 6 Hz), 4.1 (1H, d, *J =* 10 Hz), 4.0 (1H, dd, *J =* 3.5, 10 Hz), 3.9 (1H, q, *J =* 6 Hz), 3.6 (1H, m).^13^C-NMR (100 MHz, CDCl_3_) ppm 151.7, 136.9, 128.7, 123.4, 118.2, 85.2, 81.5, 78.5, 73.2, 73.1, 71.9. HRMS C_13_H_15_NO_5_ [M + Na]^+^ requires 288.0842, found 288.0891.

*Isosorbide-2-butylcarbamate* (**6c**)*.* Yield 100%. ^1^H-NMR (400 MHz, CDCl_3_) δ 5.2 (1H, d, *J =* 3 Hz), 4.8 (1H, br s), 4.6 (1H, t, *J =* 5 Hz), 4.5 (1H, d, *J =* 3 Hz), 4.3 (1H, q, *J =* 6 Hz), 4.1 (1H, d, *J =* 10 Hz), 4.0 (1H, dd, *J =* 3.5, 10 Hz), 3.9 (1H, q, *J =* 6 Hz), 3.6 (1H, m), 3.2 (2H, m), 1.6 (2H, m), 1.4 (2H, m) 0.95 (3H, t, *J =* 7.5 Hz). ^13^C-NMR (100 MHz, CDCl_3_) ppm. 154.6, 85.3, 81.5, 78.1, 73.4, 73.1, 71.9, 40.6, 31.4, 19.4, 13.3. HRMS C_11_H_20_NO_5_ [M + H]^+^ requires 246.1336, found 248.1366.

*Isosorbide-2-ethylcarbamate* (**6d**)*.* Yield 100%.^1^H-NMR (400 MHz, CDCl_3_) δ 5.2 (1H, d, *J =* 3 Hz), 4.6 (1H, t, *J =* 5 Hz), 4.5 (1H, d, *J =* 3 Hz), 4.3 (1H, q, *J =* 6 Hz), 4.1 (1H, d, *J =* 10 Hz), 4.0 (1H, dd, *J =* 3.5, 10 Hz), 3.9 (1H, q, *J =* 6 Hz), 3.6 (1H, m), 3.2 (2H, t, *J =* 6 Hz), 2.5 (1H, br s), 1.1 (3H, t, *J =* 7.5 Hz). ^13^C-NMR (100 MHz, CDCl_3_) ppm 154.5, 85.3, 81.4, 78.0, 73.4, 73.0, 71.9, 35.5, 14.7. HRMS C_9_H_16_NO_5_ [M + H]^+^ requires 218.1083, found 218.1104.

#### 3.1.3. General Procedure for Synthesis of Lipoic Acid Esters (GP3) 

*Isosorbide-2-benzylcarbamate-5-lipoic acid ester* (**7a**)*.* Isosorbide-2-benzylcarbamate (0.3 g, 1.07 mmol), lipoic acid (0.266 g, 1.28 mmol, 1.2 eq.) and dimethyl amino pyridine (0.157, 1.28 mmol, 1.2 eq.) were dissolved in anhydrous DCM (150 mL) and stirred for 10 min at room temperature in the presence of 3 Å molecular sieves. *N*-(3-Dimethylaminopropyl)-*N*-ethylcarbodiimide hydrochloride (EDCI, 0.245 g, 1.28 mmol, 1.2 eq.) was then added and the reaction mixture was stirred in the dark for 12 h, filtered and concentrated under reduced vacuum. The concentrated reaction mixture was purified by column chromatography using silica gel without further preparation. Methanol (5%) in chloroform was used as eluent to give (**7a**) as a pale yellow solid (0.145 g, 31%). ^1^H-NMR (CDCl_3_) δ ppm: 7.34 (5H, m, n), 5.15 (2H, m, m), 4.82 (1H, d, *J* = 5 Hz, k), 4.53 (1H, d, *J* = 4.5 Hz), 4.40 (2H, d, *J =* 6 Hz), 4.05–3.95 (4H, m,), 3.6 (1H, m), 3.15 (2H, m,), 2.55 (1H, m), 2.47 (2H, m), 1.94 (1H, m), 1.68 (4H, m), 1.52 (2H, m). ^13^C-NMR (CDCl_3_): 172.4, 154.8, 137.6, 128.3, 127.2, 127.1, 85.6, 80.2, 78.0, 76.8, 73.2, 69.9, 55.9, 44.7, 39.8, 38.0, 34.1, 33.4, 33.3, 30.5, 28.2, 25.1. HRMS C_22_H_29_NO_6_S_2_ [M + Na]^+^ requires 488.1536 found 488.1531.

*Isosorbide-2-phenylcarbamate-5-lipoic acid ester* (**7b**)*.* Yield 45%. Pale yellow crystals. ^1^H-NMR (CDCl_3_) δ ppm: 7.4 (4H, m), 7.1, (1H, t, *J =* 7.5 Hz), 5.15 (2H, m), 4.82 (1H, d, *J =* 5 Hz), 4.53 (1H, d, *J =* 4.5 Hz), 4.05–3.95 (4H, m), 3.65 (1H, m), 3.15 (2H, m), 2.55 (1H, m), 2.47 (2H, m), 1.94 (1H, m), 1.68 (2H, m). ^13^C-NMR (CDCl_3_): 172.4, 151.7, 136.9, 128.7, 123.4, 118.2, 85.2, 81.5, 78.5, 73.2, 73.2, 71.9, 55.9, 39.8, 38.0, 34.1, 33.4, 28.2, 25.1. HRMS: C_21_H_27_NO_6_S_2_ [M + H]^+^ requires 454.1353, found 454.1358.

*Isosorbide-2-butylcarbamate-5-lipoic acid ester* (**7c**). Yield 60%. ^1^H-NMR (CDCl_3_) δ ppm: 5.15 (2H, m), 4.82 (1H, d, *J =* 5 Hz), 4.53 (1H, d, *J =* 4.5 Hz), 4.05–3.93 (4H, m), 3.60 (1H, m), 3.25–3.15 (4H, m), 2.55 (1H, m), 2.47 (2H, m), 1.94 (1H, m), 1.68 (4H, m), 1.59 (2H, m), 1.52 (2H, m), 1.45 (2H, m), 0.95 (3H, t, *J =* 7.5 Hz). ^13^C-NMR (CDCl_3_): 172.4, 154.6, 85.3, 81.5, 78.1, 73.4, 73.1, 71.9, 55.8, 40.6, 39.8, 38.0, 34.1, 33.4, 31.4, 28.2, 25.1, 19.4, 13.3. HRMS: C_19_H_31_NO_6_S_2_ [M + H]^+^ requires 434.1666, found 434.1670.

*Isosorbide-2-ethylcarbamate-5-lipoic acid ester* (**7d**). Yield 56%. ^1^H-NMR (CDCl_3_) δ ppm: 5.15 (2H, m), 4.82 (1H, d, *J =* 5 Hz), 4.53 (1H, d, *J =* 4.5 Hz), 4.05–3.93 (4H, m), 3.60 (1H, m) 3.25 (2H, m), 3.15 (2H, m), 2.55 (1H, m), 2.47 (2H, m), 1.94 (1H, m), 1.68 (4H, m), 1.52, (2H, m), 1.10 (3H, t, *J* = 7.5 Hz). ^13^C-NMR (MHz 100.16, CDCl_3_): 172.4, 85.3, 81.4, 78.0, 73.4, 73.0, 71.9, 55.9, 39.8, 38.0, 35.6, 34.1, 33.4, 28.2, 25.1, 14.7. HRMS: C_17_H_27_NO_6_S_2_ [M + H]^+^ requires 406.1353, found 406.1358.

#### 3.1.4. General Procedure for Synthesis of Carbamate Ferulates **8a**–**d** (GP4)

*Isosorbide-2-benzylcarbanate-5-ferulate* (**8a**). Isosorbide-2-benzylcarbamate (0.3 g, 1.07 mmol), ferulic acid (0.266 g, 1.28 mmol, 1.2 eq.) and dimethyl amino pyridine (0.157, 1.28 mmol, 1.2 eq.) were dissolved in anhydrous DCM (150 mL) and stirred for 10 min at room temperature in the presence of 3 Å molecular sieves. *N*-(3-Dimethylaminopropyl)-*N*-ethylcarbodiimide hydrochloride (EDCI, 0.245 g, 1.28 mmol, 1.2 eq.) was then added and the reaction mixture was stirred in the dark for 12 h, filtered and concentrated under vacuum. The concentrated reaction mixture was purified by column chromatography using silica gel without further preparation. Methanol (5%) in chloroform was used as eluent to give (**6**) as a pale yellow solid (0.145 g, 31%). ^1^H-NMR δ (400 MHz, CDCl_3_): 7.56 (1H, d, *J* = 16.1 Hz), 7.40−7.23 (m, 5H), 7.0–6.83 (3H, m) 6.22 (1H, d, *J* = 16.1 Hz), 5.84 (1H, s), 5.22 (d, 1H, *J* = 3.01 Hz), 5.17 (m, 1H), 4.61 (t, 1H, *J* = 4.77 Hz), 4.51 (d, 1H, *J* = 4.01 Hz), 4.38 (d, 2H, *J* = 6.03 Hz), 4.31 (m, 1H), 4.10 (d, 1H, *J* = 10.45 Hz), 4.00 (dd, 1H, *J* = 3.51, 10.54 Hz), 3.90 (4H, m), 3.57 (dd, 1H, *J* = 5.53, 8.54 Hz), 2.67 (d, 1H, *J* = 7.03 Hz). ^13^C-NMR ppm (100.16 MHz., CDCl_3_): 44.7, 55.8, 71.8, 73.0, 73.4, 78.4, 81.5, 85.3, 109.3, 114.6, 114.8, 122.8, 126.6, 127.1, 127.2, 128.3, 137.6, 144.8, 146.6, 147.8,9 154.7, 167.6. HRMS: C_24_H_25_NO_8_ [M + H]^+^ requires 456.1653, found 456.1670.

*Isosorbide-2-phenylcarbanate-5-ferulate* (**8b**). Yield 65%. ^1^H-NMR (400 MHz, CDCl_3_) δ ppm: 7.56 (1H, d, *J =* 16.1 Hz), 7.41 (4H, m), 7.11 (1H, t, *J =* 7.5 Hz), 7.0–6.85 (3H, m), 6.80 (1H, br s), 6.22 (1H, d, *J =* 16.1 Hz), 5.84, (1H, s) 5.31 (1H, d, *J =* 3 Hz), 4.72 (1H, t, *J =* 5 Hz), 4.53 (1H, d, *J =* 3 Hz), 4.33 (1H, q, *J =* 6 Hz), 4.10 (1H, d, *J =* 10 Hz), 4.00 (1H, dd, *J =* 3.5, 10 Hz), 3.95 (1H, s), 3.90 (1H, q, *J =* 6 Hz), 3.61 (1H, m). ^13^C-NMR (100 MHz, CDCl_3_) ppm. 167.6, 151.7, 147.8, 146.6, 144.8, 136.9, 128.7, 126.6, 123.4, 118.2, 114.8, 114.7, 109.6 85.2, 81.5, 78.5, 73.2, 73.1, 71.9, 55.8. HRMS: C_23_H_23_NO_8_ [M + H]^+^ requires 442.1496, found 442.1495.

*Isosorbide-2-butylcarbanate-5-ferulate* (**8c**). Yield 60%. ^1^H-NMR (400 MHz, CDCl_3_) δ ppm: 7.60 (1H, d, *J =* 16.1 Hz), 7.12 – 6.90 (3H, m), 6.25 (1H, d, *J =* 16.1 Hz), 5.88 (1H, s), 5.23 (1H, d, *J =* 3 Hz), 4.82 (1H, br s), 4.61 (1H, t, *J =* 5 Hz), 4.53 (1H, d, *J =* 3 Hz), 4.39 (1H, q, *J =* 6 Hz), 4.11 (1H, d, *J =* 10 Hz), 4.01 (1H, dd, *J =* 3.5, 10 Hz), 3.91 (2H, m), 3.60 (1H, m), 3.21 (2H, m), 1.61 (2H, m), 1.40 (2H, m), 0.95 (3H, t, *J =* 7.5 Hz). ^13^C-NMR (100.16 MHz, CDCl_3_) ppm. 167.8, 154.6, 147.7, 146.7, 144.9, 126.5, 114.9, 114.6, 109.6, 85.3, 81.5, 78.1, 73.4, 73.1, 71.9, 55.79, 40.6, 31.4, 19.4, 13.3. HRMS: C_21_H_27_NO_8_ [M + H]^+^ requires 422.1809, found 422.1799.

*Isosorbide-2-ethylcarbanate-5-ferulate* (**8d**). Yield 60%. ^1^H-NMR (CDCl_3_) δ ppm: 7.56 (1H, d, *J =* 16.1 Hz), 7.09–6.83 (3H, m), 6.22 (1H, d, *J =* 16.1 Hz), 5.87 (1H, s) 5.21 (1H, d, *J =* 3 Hz), 4.60 (1H, t, *J =* 5 Hz), 4.53 (1H, d, *J =* 3 Hz), 4.33 (1H, q, *J =* 6 Hz), 4.06 (1H, d, *J =* 10 Hz), 4.0 (1H, dd, *J =* 3.5, 10 Hz), 3.91 (2H, m), 3.60 (1H, m), 3.21 (2H, t, *J =* 6 Hz), 2.51 (1H, br s), 1.11 (3H, t, *J =* 7.5 Hz). ^13^C-NMR (100 MHz, CDCl_3_) ppm 167.6, 154.5, 147.9, 146.6, 144.8, 126.6, 114.8, 114.7, 109.6, 85.3, 81.4, 78.0, 73.4, 73.0, 71.9, 55.8, 35.5, 14.7. HRMS: C_19_H_23_NO_8_ [M + H]^+^ requires 394.1496, found 394.1501.

### 3.2. Biochemical Studies

#### 3.2.1. Ellman Assay for Measuring Inhibition of Cholinesterase Activity

Cholinesterase activity was measured using the Ellman spectrophotometric method. *Hu*BuChE activity was measured in replicate samples using a 96-well plate reader. The total volume of test solution in each well was 250 μL. This consisted of 25 μL of plasma solution, 150 μL of phosphate buffer pH 8.0, 25 μL of DTNB solution (0.5 mM), and 25 μL of acetonitrile:distilled water (1:1). The 96-well plate was incubated for 30 min before 25 μL of butyrylthiocholine iodide (BTCI) solution (0.5 mM) was added, and the reaction was measured at 405 nm over 5 min using an Anthos bt2 plate reader. For the determination of AChE activity, 25 μL of electric eel AChE solution and 25 μL of acetylthiocholine iodide (ATCI) solution were used instead of the plasma solution and BTCI solution. For determination of enzyme inhibition, 25 μL of an inhibitor solution was added to the test solution instead of the acetonitrile:water (1:1) solution. Inhibitors were added 30 min before determination of remaining enzyme activity. IC_50_ values were calculated using GraphPad Prism 4.02 software (GraphPad Software Inc., La Jolla, CA, USA).

#### 3.2.2. Inhibitor Kinetics

In order to monitor enzyme carbamylation over time, a 96-well plate was used, with all assay mixtures being made up to a final concentration of 250 μL for monitoring of absorbance at 412 nm. At time 0, 150 μL of inhibitor, at various concentrations, was added to an equal volume of enzyme. Thereafter, at successive time points, 50 μL aliquots were removed from these wells and added to wells containing 200 μL DTNB and BTCI in phosphate buffer pH 8.0. Hydrolysis of substrate was monitored by examining the changes in absorbance in each well at 412 nm over the following 30 s. Negative controls were run alongside the test solutions to take account of non-enzymatic hydrolysis, while positive controls gauged the rate of the uninhibited reaction. At least two replicate wells were run for each concentration of inhibitor. Plots of percentage inhibition over time were prepared using GraphPad Prism 4.03. A first order inhibition constant, *k_obs_* was obtained for each concentration of inhibitor by non-linear regression using an exponential decay function.

#### 3.2.3. MTT Assay and Neuroprotection of HT-22 Neurons

This test was performed as reported before. Briefly, HT-22 cells were seeded in 96-well plates at a density of 5 × 10^3^ per well and cultured for 24 h. Subsequently, cells were incubated for another 24 h either with medium or test compounds with potential cytoprotective activity either in the absence (to test for toxic effects) or the presence (to test for the protective potential against glutamate induced oxidative stress) of 5 mM glutamate. MTT solution (4 mg/mL in PBS) was diluted 1:10 with medium and the mixture was added to the wells after removal of the previous medium. The plates were then incubated for another 3 h. Then, the supernatant was removed and 100 µL of lysis buffer (10% SDS, pH 4.1) were added to the wells. Absorbance at 560 nm was determined on the next day using a multiwell plate photometer (Spectra Fluor Plus, Tecan, Crailsheim, Germany). The results of these cell viability assays were expressed as percentages relative to untreated control cells. All compounds were dissolved in DMSO and diluted with fresh medium, with the DMSO concentration always below 0.1% (*v*/*v*). 

#### 3.2.4. Statistics

Data of all MTT tests were expressed as means ± SD of at least three independent experiments (in quadruplicates). Data were subjected to one-way ANOVA followed by Dunnett’s multiple comparison post-test using GraphPad Prism 5 Software. (Levels of significance ** *p* < 0.01; *** *p* < 0.001).

#### 3.2.5. ORAC Assay

Compounds were assessed for antioxidant capacity using the standard ORAC assay performed as described previously [29,30,31].

## 4. Conclusions

Ferulate and lipoate esters as potential antioxidants were successfully introduced into the isosorbide-2-carbamate scaffold, which has previously been shown to be useful in preparation of highly potent and selective cholinesterase inhibitors. The resultant compounds were shown to inhibit BuChe with potency and selectivity. Moreover, in both lipoate and ferulate series, the integrated anti-oxidant promoted carbamylation or increased potency relative to the unsubstituted compounds by enforcing hydrophobic interactions with the cholinesterase active site. Compound **7a** is a potent (IC_50_ 150 nM) and selective inhibitor (667 fold) of BuChe over AChE, and, in the neuroprotection assay, it was efficacious in preventing glutamate induced cytotoxicity in a murine hippocampal cell line in the range of 10–25 µM. The ferulate esters and parent compound were not active in this model. This work shows the versatility of the isosorbide scaffold in the design of esterase directed compounds.

## Figures and Tables

**Table 1:** Carbamate–antioxidant conjugate inhibitory data for AChE and BuChE and selectivity (*n =* 3).

Cpd	R	R’	BChE IC_50_ µM (pIC_50_ ± SEM)	AChE IC_50_ μM (%I at 100 μM)	Selectivity ^†^
**7a**	Bn	Lipoate	0.15 (2.17 ± 0.03)	(30.75)	>667
**7b**	Ph	Lipoate	13.30 (4.12 ± 0.11)	(18.75)	>7.5
**7c**	Bu	Lipoate	0.17 (2.32 ± 0.04)	(68.70)	
**7d**	Et	Lipoate	5.71 (3.76 ± 0.92)	(84.10)	
**8a**	Bn	Ferulate	1.82 (3.21 ± 0.02)	(25.20)	>55
**8b**	Ph	Ferulate	29.16 (4.42 ± 0.06)	27.1 ± 11.3 μM	0.92
**8c**	Bu	Ferulate	0.43 (2.59 ± 0.05)	ND ^††^	
**8d**	Et	Ferulate	95.03 (1.98 ± 0.89)	ND	
**6a**	Bn	H	25.78 (1.41 ± 0.03)	37.5 ± 7.4 μM	1.45
**6b**	Ph	H	>100 μM	(25.30)	
**6c**	Bu	H	27.98 (1.45 ± 0.02)	(30.0)	3.57
**6d**	Et	H	46.86 (1.67 ± 0.03)	(22.0)	2.13

^†^ This was estimated from IC_50_ ratios for AChE/IC_50_ for BuChE: a value of 100 µM was used for AChE where % inhibition at 100 µM was <50%. ^††^ Not significantly different from zero.

## References

[1] Bertram L., Tanzi R.E. (2005). The genetic epidemiology of neurodegenerative disease. J. Clin. Investig..

[2] Bartus R.T., Dean R.L., Beer B., Lippa A.S. (1982). The cholinergic hypothesis of geriatric memory dysfunction. Science.

[3] Deardorff W.J., Feen E., Grossberg G.T. (2015). The use of cholinesterase inhibitors across all sages of Alzheimer’s Disease. Drugs Aging.

[4] Tan C.C., Yu J.T., Wang H.F., Tan M.S., Meng X.F., Wang C., Jiang T., Zhu X.C., Tan L. (2014). Efficacy and safety of donepezil, galantamine, rivastigmine, and memantine for the treatment of Alzheimer’s disease systematic review and meta-analysis. J. Alzheimers Dis..

[5] Brus B., Košak U., Turk S., Pišlar A., Coquelle N., Kos J., Stojan J., Colletier J.P., Gobec S. (2014). Discovery, biological evaluation, and crystal structure of a novel nanomolar selective butyrylcholinesterase inhibitor. J. Med. Chem..

[6] Huang G., Kling B., Darras F.H., Heilmann J., Decker M. (2014). Identification of a neuroprotective and selective butyrylcholinesterase inhibitor derived from the natural alkaloid evodiamine. Eur. J. Med. Chem..

[7] Chen X., Tikhonova I.G., Decker M. (2011). Probing the mid-gorge of cholinesterases with spacer-modified bivalent quinazolinimines leads to highly potent and selective butyrylcholinesterase inhibitors. Bioorg. Med. Chem..

[8] Maurice T., Strehaiano M., Siméon N., Bertrand C., Chatonnet A. (2016). Learning performances and vulnerability to amyloid toxicity in the butyrylcholinesterase knockout mouse. Behav. Brain Res..

[9] Greig N.H., Utsuki T., Ingram D.K., Wang Y., Pepeu G., Scali C., Yu Q.S., Mamczarz J., Holloway H.W., Giordano T. (2005). Selective butyrylcholinesterase inhibition elevates brain acetylcholine, augments learning and lowers Alzheimer beta-amyloid peptide in rodent. Proc. Natl. Acad. Sci. USA.

[10] Mesulam M.M., Guillozet A., Shaw P., Levey A., Duysen E.G., Lockridge O. (2002). Acetylcholinesterase knockouts establish central cholinergic pathways and can use butyrylcholinesterase to hydrolyze acetylcholine. Neuroscience.

[11] Montanari S., Bartolini M., Neviani P., Belluti F., Gobbi S., Pruccoli L., Tarozzi A., Falchi F., Andrisano V., Miszta P. (2015). Multitarget Strategy to Address Alzheimer’s Disease: Design, Synthesis, Biological Evaluation, and Computational Studies of Coumarin-Based Derivatives. ChemMedChem.

[12] Decker M. (2007). Recent advances in the development of hybrid molecules/designed multiple compounds with antiamnesic properties. Mini Rev. Med. Chem..

[13] Koufaki M., Detsi A., Kiziridi C. (2009). Multifunctional lipoic acid conjugates. Curr. Med. Chem..

[14] Perry G., Cash A.D., Smith M.A. (2002). Alzheimer Disease and Oxidative Stress. J. Biomed. Biotechnol..

[15] Park S., Karunakaran U., Jeoung N.H., Jeon J.H., Lee I.K. (2014). Physiological effect and therapeutic application of alpha lipoic acid. Curr. Med. Chem..

[16] Decker M., Kraus B., Heilmann J. (2008). Design, synthesis and pharmacological evaluation of hybrid molecules out of quinazolinimines and lipoic acid lead to highly potent and selective butyrylcholinesterase inhibitors with antioxidant properties. Bioorg. Med. Chem..

[17] Rosini M., Simoni E., Bartolini M., Tarozzi A., Matera R., Milelli A., Hrelia P., Andrisano V., Bolognesi M.L., Melchiorre C. (2011). Exploiting the lipoic acid structure in the search for novel multitarget ligands against Alzheimer’s disease. Eur. J. Med. Chem..

[18] Sultana R. (2012). Ferulic acid ethyl ester as a potential therapy in neurodegenerative disorders. Biochim. Biophys. Acta.

[19] Mori T., Koyama N., Guillot-Sestier M.V., Tan J., Town T. (2013). Ferulic acid is a nutraceutical β-secretase modulator that improves behavioral impairment and Alzheimer-like pathology in transgenic mice. PLoS ONE.

[20] Yan J.J., Jung J.S., Kim T.K., Hasan A., Hong C.W., Nam J.S., Song D.K. (2013). Protective effects of ferulic acid in amyloid precursor protein plus presenilin-1 transgenic mouse model of Alzheimer disease. Biol. Pharm. Bull..

[21] Benchekroun M., Bartolini M., Egea J., Romero A., Soriano E., Pudlo M., Luzet V., Andrisano V., Jimeno M.L., López M.G. (2015). Novel tacrine-grafted Ugi adducts as multipotent anti-Alzheimer drugs: A synthetic renewal in tacrine-ferulic acid hybrids. ChemMedChem.

[22] Carolan C.G., Dillon G.P., Gaynor J.M., Reidy S., Ryder S.A., Khan D., Marquez J.F., Gilmer J.F. (2008). Isosorbide-2-carbamate Esters: Potent and Selective Butyrylcholinesterase Inhibitors. J. Med. Chem..

[23] Dillon G.P., Gaynor J.M., Khan D., Carolan C.G., Ryder S.A., Marquez J.F., Reidy S., Gilmer J.F. (2010). Isosorbide-based cholinesterase inhibitors; replacement of 5-ester groups leading to increased stability. Bioorg. Med. Chem..

[24] Carolan C.G., Dillon G.P., Khan D., Ryder S.A., Gaynor J.M., Reidy S., Marquez J.F., Jones M., Holland V., Gilmer J.F. (2010). Isosorbide-2-benzyl carbamate-5-salicylate, a peripheral anionic site binding subnanomolar selective butyrylcholinesterase inhibitor. J. Med. Chem..

[25] Carolan C.G., Gaynor J.M., Dillon G.P., Khan D., Ryder S.A., Reidy S., Gilmer J.F. (2008). Novel isosorbide di-ester compounds as inhibitors of acetylcholinesterase. Chem. Biol. Interact..

[26] Kling B., Bücherl D., Palatzky P., Kling B., Bücherl D., Palatzky P., Matysik F.M., Decker M., Wegener J., Heilmann J. (2014). Flavonoids, flavonoid metabolites, and phenolic acids inhibit oxidative stress in the neuronal cell line HT-22 monitored by ECIS and MTT assay: A comparative study. J. Nat. Prod..

[27] Tan S., Wood M., Maher P. (1998). Oxidative stress induces a form of programmed cell death with characteristics of both apoptosis and necrosis in neuronal cells. J. Neurochem..

[28] Ishige K., Schubert D., Sagara Y. (2001). Flavonoids protect neuronal cells from oxidative stress by three distinct mechanisms. Free Radic. Biol. Med..

[29] Dávalos A., Gómez-Cordovés C., Bartolomé B. (2004). Extending applicability of the oxygen radical absorbance capacity (ORAC-fluorescein) assay. J. Agric. Food Chem..

[30] Vogel S., Ohhmayer S., Brunner G., Heilmann J. (2008). Natural and non-natural prenylated chalcones: Synthesis, cytotoxicity and anti-oxidative activity. Bioorg. Med. Chem..

[31] Fang L., Kraus B., Lehmann J., Heilmann J., Zhang Y., Decker M. (2008). Design and synthesis of tacrine-ferulic acid hybrids as multi-potent anti-Alzheimer drug candidates. Bioorg. Med. Chem. Lett..

